# Population screening for colorectal cancer by flexible sigmoidoscopy or CT colonography: study protocol for a multicenter randomized trial

**DOI:** 10.1186/1745-6215-15-97

**Published:** 2014-03-28

**Authors:** Daniele Regge, Gabriella Iussich, Carlo Senore, Loredana Correale, Cesare Hassan, Alberto Bert, Stefania Montemezzi, Nereo Segnan

**Affiliations:** 1 Radiology Unit, Institute for Cancer Research and Treatment, FPO, Strada Provinciale 142, Candiolo 10060, Italy; 2 CPO Piemonte and AO ‘City of Health and Science,’ SC Epidemiologia dei Tumori, Turin, Italy; 3 im3D SpA, Turin, Italy; 4 Department of Radiological Sciences Oncology and Pathology, University of Rome La Sapienza, Rome, Italy; 5 Radiology Unit, Azienda Ospedaliera di Verona, Verona, Italy

**Keywords:** Colorectal cancer, Screening, Flexible sigmoidoscopy, CT colonography, Computer-aided detection

## Abstract

**Background:**

Colorectal cancer (CRC) is the second most prevalent type of cancer in Europe. A single flexible sigmoidoscopy (FS) screening at around the age of 60 years prevents about one-third of CRC cases. However, FS screens only the distal colon, and thus mortality from proximal CRC is unaffected. Computed tomography colonography (CTC) is a highly accurate examination that allows assessment of the entire colon. However, the benefit of CTC testing as a CRC screening test is uncertain. We designed a randomized trial to compare participation rate, detection rates, and costs between CTC (with computer-aided detection) and FS as primary tests for population-based screening.

**Methods/Design:**

An invitation letter to participate in a randomized screening trial comparing CTC versus FS will be mailed to a sample of 20,000 people aged 58 or 60 years, living in the Piedmont region and the Verona district of Italy. Individuals with a history of CRC, adenomas, inflammatory bowel disease, or recent colonoscopy, or with two first-degree relatives with CRC will be excluded from the study by their general practitioners. Individuals responding positively to the invitation letter will be then randomized to the intervention group (CTC) or control group (FS), and scheduled for the screening procedure. The primary outcome parameter of this part of the trial is the difference in advanced neoplasia detection between the two screening tests. Secondary outcomes are cost-effectiveness analysis, referral rates for colonoscopy induced by CTC versus FS, and the expected and perceived burden of the procedures. To compare participation rates for CTC versus FS, 2,000 additional eligible subjects will be randomly assigned to receive an invitation for screening with CTC or FS. In the CTC arm, non-responders will be offered fecal occult blood test (FOBT) as alternative screening test, while in the FS arm, non-responders will receive an invitation letter to undergo screening with either FOBT or CTC. Data on reasons for participation and non-participation will also be collected.

**Discussion:**

This study will provide reliable information concerning benefits and risks of the adoption of CTC as a mass screening intervention in comparison with FS. The trial will also evaluate the role of computer-aided detection in a screening setting.

**Trial registration:**

ClinicalTrials.gov Identifier: NCT01739608

## Background

Colorectal cancer (CRC) is the second leading cause of cancer-related death in Europe and the USA [1]. It is the most frequently diagnosed cancer in Europe for both genders combined, with more than 400,000 new cases and more than 200,000 deaths in 2008 [1]. About 46,000 incident cases, 267,000 prevalent cases, and 16,000 deaths from CRC are estimated in Italy for the year 2005 [2]. The lifetime risk of CRC in western countries is about 5%. Population screening in asymptomatic individuals at average risk for CRC reduces mortality, both through the detection of malignancies at earlier, more treatable stages, and through the identification and removal of adenomatous polyps (pre-cancerous lesions that may evolve to CRC) [3-9]. In addition, given the increase in the costs of CRC treatment, screening has actually become cost-saving [10]. There are several tests currently available for screening in the general population, including stool tests, such as variants of the fecal occult blood test (FOBT), and structural examinations such as flexible sigmoidoscopy (FS), colonoscopy, and CT colonoscopy (CTC). Each screening strategy has its advantages and drawbacks.

The simplest and best-evaluated available screening method is the FOBT, which is relatively inexpensive and non-invasive, but less accurate than structural examinations. The guaiac-based FOBT (gFOBT) may reduce cancer mortality by up to 20% if offered biennially [4,5,11], and possibly more if offered annually [8]. The newer immunochemical FOBT (iFOBT) is considered to perform better than gFOBT in detecting advanced neoplasia [12]. The disadvantages of FOBT include its low sensitivity for adenoma and the requirement for frequent testing, which may limit compliance and thereby effectiveness. Furthermore, repeat testing leads to high positivity rates.

FS is an endoscopic procedure, in which the distal 40 to 60 cm of the colon is inspected. Total colonoscopy is advised in cases of positive findings. FS is less invasive than colonoscopy, and requires only a simple bowel preparation of a single enema within 2 hours prior to the procedure. Three European randomized control trials (RCTs) on FS have been performed [9,13,14]. In the UK, one-time screening with FS significantly reduced the incidence of CRC (by 26%) and associated mortality (by 36%) [13]. In Italy, an 18% reduction in incidence and a non-significant 22% reduction in mortality were reported [9], whereas in Norway, no benefit was observed after 7 years of follow-up [15]. A disadvantage of FS is that it does not examine the proximal colon, but distal findings are used to select a higher-risk group for colonoscopy. Another consideration is that its sensitivity depends on the varied experience of the examiners, that has a major impact on the adequacy of mucosal inspection.

Colonoscopy and CTC are structural examinations allowing inspection of the complete colon, and enabling early detection of advanced adenomas (that is,, adenomas ≥10 mm or with unfavorable histological features) and CRC [16]. Although colonoscopy is the most complete endoscopic procedure available for CRC screening [17,18], direct evidence about its effectiveness, complications, and acceptability among individuals at average risk of CRC is still not adequate to justify its use for population screening [19]. Observational evidence suggests that colonoscopy might not be as effective in the proximal colon as in the other segments of the colon and rectum [20,21]. Furthermore, colonoscopy capacity is a limiting factor for its widespread use as a primary screening test.

CTC may represent a reasonable alternative for colonoscopy [16]. First, this technique has a very high sensitivity for already-developed CRC (96%) [22]. Secondly, results from a large study of asymptomatic average-risk individuals published in 2003 showed the diagnostic performance of CTC for clinically relevant polyps to be equivalent to that of colonoscopy [23]. Results from the largest screening study (over 2,500 participants) [24] showed 90% sensitivity of CTC for polyps 10 mm or larger and 86% specificity; the positive and negative predictive values were 23% and 99%, respectively. Important advantages of CTC over colonoscopy are its minimally invasive nature (only a small-caliber flexible rectal catheter is needed for colonic distension) and the use of limited bowel preparation [25,26]. The risk of complications from CTC is extremely low, particularly in asymptomatic individuals [27-30]. CTC with limited bowel preparation has a lower burden and is preferred by patients compared with CTC with full cathartic preparation [31,32]. Potentially, the addition of CTC to CRC screening options could have a marked effect on current low adherence rates, likely in a cost-effective manner [33-35]. Indeed, results from a recent trial [25] on the use of colonoscopy versus CTC for population-based screening for CRC showed that participation with CTC was substantially higher than with colonoscopy, and thus led to similar advanced neoplasia detection rates. Disadvantages of CTC include the exposure of individuals to ionizing radiation. However, the chances of radiation-induced malignancy are considered very low, especially when a low-dose protocol is used. Furthermore, screening with CTC requires subsequent colonoscopy if lesions are detected. A high referral rate to colonoscopy might increase examination costs. CTC displays the abdominal organs external to the colon. The prevalence of extracolonic diseases requiring further investigation is not negligible, occurring in approximately 6% of the individuals in asymptomatic average-risk populations [25,36]. It is generally agreed that relevant extracolonic findings should be reported [37]. However, although the detection of relevant extracolonic diseases at CTC could be beneficial [38], the risks and costs associated with false-positive results and inconsequential findings may be substantial [39].

Data concerning the neoplasia yield and the acceptability of CTC compared with colonoscopy have been recently reported from the CoCos trial [25], but no information is available concerning the performance of CTC compared with the recommended screening tests, namely, iFOBT and FS. In European countries where national screening programs are ongoing, national policy makers would require comparative data concerning the performance of CTC (as of any other new screening test) assessed against existing strategies before considering the introduction of this test as an available screen for average-risk individuals. A RCT, the SAVE Trial, has recently been designed and funded in Italy to compare CTC and iFOBT in a screening setting [40].

We are currently conducting a multicenter RCT to compare the performance of CTC and FS in the context of the population-based screening programs offering FS once in a person’s lifetime in the Piedmont region and the province of Verona in Italy. These organized programs were started in 2004, targeting all men and women, aged 58 years (in Piedmont region) or 60 years (in Verona) who are at average risk of CRC. Individuals who do not respond to the FS invitation are offered iFOBT as alternative.

Several issues need however to be addressed before the implementation of a program using CTC as a primary CRC screening test. First, the local availability and expertise of radiologists may affect the feasibility of CTC screening. The use of information technology (IT) infrastructures may allow subjects to undergo CTC at their nearest imaging center, while test interpretation could take place at a centralized level, requiring a smaller number of readers. Implementing telediagnosis should thus ensure high reporting quality, provided that highly qualified radiologists, certified to report CTC, are selected. The local availability of radiological centers could also favor greater participation of individuals in the screening programs.

Secondly, it is widely recognized that the interpretation of CTC is challenging, probably more so in a low-prevalence population. The need to view a large number of images to detect a small number of clinically significant lesions, the subtle nature of many radiological characteristics of colonic lesions, and radiologist fatigue or distraction may lead to an undesirable rate of false-negative findings [41,42]. Computer-aided detection (CAD) has the potential to improve the cost-effectiveness of CTC by increasing detection of clinically significant lesions and/or reducing reporting times [43]. CAD as a second reader (that is, CAD is applied after a complete and unaided assessment) has been shown to increase reading sensitivity, albeit at the cost of increasing reading times [44-47]. This increase in reading times is generally undesirable if a large number of cases must be read sequentially, as occurs for population screening. A potentially more time-efficient paradigm is first-reader CAD, in which the reader’s interpretation is restricted to CAD prompts alone [48]. However, detection of colonic findings typically not targeted by CAD systems, such as masses or atypical lesions, poses challenges [49].

The limitation of CAD for mass detection provides motivation for double-reading first-reader CAD (DR FR-CAD paradigm in which first-reader CAD is followed by a rapid two-dimensional review of unprompted areas of the colon, searching for masses or larger lesions missed by CAD [50]. The addition of the human component to first-reader CAD as a rapid control for eventual CAD errors may overcome the issues related to automatic detection. We hypothesized that DR FR-CAD could play a key role in CTC screening by improving detection sensitivity compared with unassisted reading, and by shortening interpretation time (while maintaining equally high sensitivity) compared with second-reader CAD. Thus we performed two preliminary studies [50,51] to investigate the feasibility of using DR FR-CAD as a possible reading strategy for CTC screening and to compare the diagnostic performance and time-efficiency with those of second-reader CAD for CRC screening [51]. Participants included in this preliminary study underwent limited bowel preparation with fecal tagging, as the available evidence showed that limited preparation was not associated with a decrease in the CTC diagnostic performance compared with full-cathartic preparation [52].

This multicenter RCT, assessing an average-risk population aged 58 to 60 years, will compare the detection rates of advanced neoplasia (advanced adenoma and CRC) of CT colonography and sigmoidoscopy, and the participation rates for the two programs. The data from our preliminary research have allowed us to define a CTC screening protocol (adopting an organizational model based on telediagnosis together with DR FR-CAD for CTC interpretation). The cost-effectiveness of this protocol will also be tested in the proposed RCT.

## Methods/Design

### Objectives

#### Primary objective

The primary objective is to compare the participation and detection rate of CTC and FS for advanced neoplasia (that is, CRC and advanced adenomas) in a population-based screening program for CRC.

### Secondary objectives

The secondary objectives are 1) to evaluate the cost-effectiveness and feasibility of CTC as a screening method for CRC; 2) to evaluate the referral rate for colonoscopy as a result of CTC versus FS; 3) to evaluate the reasons for participation and non-participation to the proposed screening strategies; 4) to evaluate the rate of complications in each screening group; and 5) to compare the expected and perceived burden of FS and CTC.

### Study population

Eligible subjects are being identified through the rosters of general practitioners (GPs). The target population includes all patients aged 58 (in the Piedmont region, Italy) or 60 years (in the Verona district, Italy) who are listed in the rosters of a random sample of GPs. In both locations, GPs are asked to exclude any individuals unable to give informed consent; those with a family (two first-degree relatives) or personal history of CRC, colorectal polyps, or inflammatory bowel disease; those who underwent a colonoscopy within the previous 5 years or a FOBT within the previous 2 years; and those with a medical condition that would preclude benefit from screening. GPs are also asked to sign the invitation letters and the reminders.

### Study design

We have designed a population-based multicenter RCT with two arms, involving six hospitals located in the Piedmont region of Italy and two hospitals in the district of Verona, Italy. To reduce bias in tests comparison, the RCT is structured in two parts: 1) study 1 is comparing the detection of CTC for advanced neoplasia with that of FS; and 2) study 2 is comparing the participation rate of CTC with that of FS. The two studies differ in their recruiting and randomization procedures. For both studies, participants will be enrolled after providing written informed consent to the study procedures, which will be performed free of charge.

**Figure 1:**
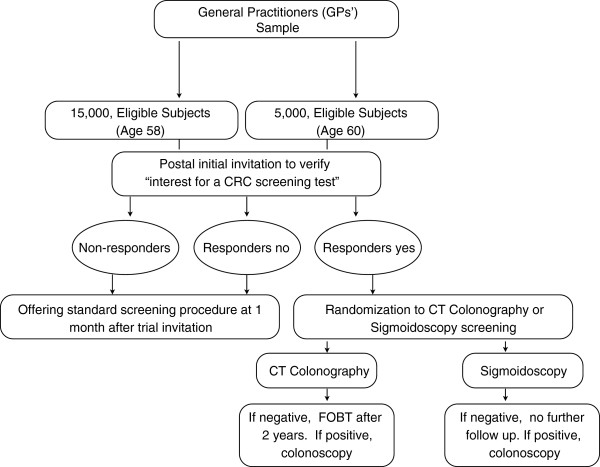
Design of the screening trial comparing detection rates of advanced neoplasia between computed tomography colonoscopy (CTC) and flexible sigmoidoscopy (FS) as primary colorectal cancer (CRC) screening tests.

In study 1, a sample of 20,000 subjects aged 58 or 60 years living, respectively, in the Piedmont region and Verona district, and who are listed in GP rosters will be sent an invitation letter to receive screening for CRC as part of a research trial (Figure 1). Individuals who agree to participate into the trial will be randomly allocated to FS or CTC screening in a 1:1 ratio. Random assignment will be performed in each center by the local coordinating unit using a computer-generated allocation algorithm. Participants will be randomly assigned on an individual basis. Non-responders or individuals who refuse to enter the trial will be invited to the standard screening program by letter 1 month after the invitation to the trial.

**Figure 2:**
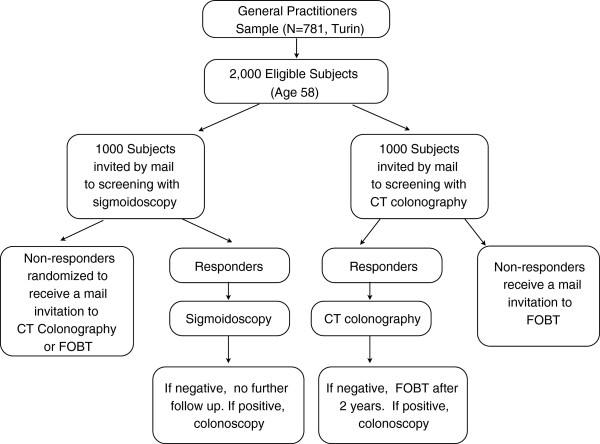
Design of the screening trial comparing participation rates between computed tomography colonoscopy (CTC) and flexible sigmoidoscopy (FS) as primary colorectal cancer (CRC) screening tests.

For study 2, an additional 2,000 individuals aged 58 years living in Turin, Italy and who are listed in GP rosters will be randomly assigned to receive an invitation for screening with CTC or FS (Figure 2). In the FS arm, non-responders will be randomized subsequently to receive an invitation for screening with CTC or FOBT at 6 months from the initial invitation. In the CTC arm, non-responders will be offered FOBT as an alternative screening test. Simple randomization will be performed by personnel of the Center for Cancer Prevention (CPO) in Piedmont, Turin, using a computer-generated allocation algorithm, with an allocation ratio of 1:1.

### Invitation procedures

Similar to our previous CRC screening trials [9,53], a specialized database will be used for the logistics of the invitation procedures. All targeted individuals will be invited to the study by mailed invitations.

In study 1, eligible individuals will be mailed a letter from their GP informing them that a CRC screening trial comparing CTC versus FS is being performed in their district, along with an information leaflet that outlines the objectives of the trial, gives a general description of the trial procedures, and describes the screening tests, their advantages, and possible risks. The mailing specifies that participation in the trial requires agreeing to be randomized to receive one of the two study screen tests. Recipients will be asked to call the screening center if they want to take part in the trial, and those who respond positively will then be randomized to either CTC or FS. The telephone operator will also give each individual detailed information on bowel preparation. In the FS arm, participants will be asked to visit their GP or the screening center to obtain the required enema kit, while in the CTC arm, individuals will be asked to visit the screening center to obtain the required bowel preparation. Non-responders and individuals who do not agree to participate in the study will receive an invitation letter to undergo standard screening 1 month after the invitation to the trial.

Differently, in study 2, the invitation letters offer a pre-booked appointment for one of the screening procedures. The mailing includes a leaflet containing a brief description of the screening procedure (CTC or FS, depending on the invitation), and its possible advantages and side effects. The leaflets are based on those used in the regional screening program. Invitees will be asked to confirm, change, or cancel their appointment. The bowel preparation procedures are the same as in study 1. Non-responders will be sent a mailed reminder 3 months after the initial invitation. In the CTC arm, individuals who do not respond to the reminder will be offered FOBT test, in accordance with the current screening procedure. In the FS arm, non-responders to the reminder will receive an invitation letter to be screened with either FOBT or CTC. The invitation letter for FOBT invites the subject to contact the GP or the screening center to obtain the kit and the necessary information for performing the test.

### Primary screening tests

#### FS

Bowel preparation will be limited to a single enema (133 ml of 22% sodium phosphate) self-administered at home 2 hours before the test. There will be no dietary restrictions recommended. Screening will be performed in hospital endoscopy units by gastroenterologists. The FS will be performed with the use of a 130 cm colonoscope. Participants with adequate bowel preparation will undergo the procedure; operators will advance the colonoscope beyond the sigmoid-descending colon junction. Although no medication (such as spasmolytic or sedative drugs) will be offered to the participants, it can be administered if the endoscopist thinks it is necessary. All polyps smaller than 10 mm detected during the FS will be removed immediately and sent for histologic evaluation. Participants with polyps 10 mm or larger as well as those detected with advanced adenomas (that is, two adenomas detected following the histological examination *as described below) will be referred for total colonoscopy. After the examination, the endoscopist will give the subject a letter that explains the results of their FS. The letter also specifies the date for colonoscopy if large polyps are detected, or for receiving the histology results if small polyps are excised during the examination.

### CTC

Individuals will be placed on a low-residue diet starting 3 days before the examination. Bowel preparation consists of administration of one sachet of a Macrogol 3350-based mild laxative (Movicol; Norgine, Milan, Italy) at each of the three main meals, starting 3 days before the examination. Two hours before the examination, participants will drink a 70 ml solution of sodium diatrizoate and meglumine diatrizoate (Gastrografin; Bayer Schering Pharma, Milan, Italy) diluted in 500 ml of water, followed by an additional 500 ml of water. Immediately before scanning, a radiologist or a trained nurse will introduce a flexible rectal catheter, and distension will be produced using an automatic carbon-dioxide insufflator (PROTOCO2L, Bracco; EZEM, Lake Success, NY, USA). n-Butyl-scopolamine will be administered in accordance with the usual practice of each participating center. The following low-dose protocol will be adopted: 120 kVp (140 kVp in obese patients with body mass index >30 kg/m^2^); 50 mA (effective) or less per second (without the use of any system for dose modulation); a rotation time of 0.5 to 0.7 seconds; a section thickness not greater than > 1.25 mm, and reconstruction interval of 1.25 mm or less. The total effective dose per study should be less than 4 mSv. Intravenous contrast medium will not be used. The radiologists and nurses involved in the study will previously have undergone a 1 day course on examination technique and quality assurance.

In the Piedmont region, CTC examinations will be performed in six different hospitals (FPO – IRCCS, Candiolo, Torino; AOU San Giovanni Battista, Torino; AO Ordine Mauriziano di Torino, AO Città della Salute e della Scienza, Torino; Ospedale di Borgomanero-Veruno, Novara; ASL di Biella, Biella) equipped with at least 16 slice scanners. CTC images will then be transferred to a centralized reading center for interpretation through a regional information communications technology (ICT) network infrastructure, using standard uncompressed Digital Imaging and Communications in Medicine (DICOM) format. Conversely, in the Verona district, examination interpretation will be performed at the same hospitals where the examinations are performed (Ospedale di Borgo Trento and Ospedale di San Bonifacio; both Verona).

For all screening sessions, image processing and interpretation will be performed using a CTC workstation with CAD software (CAD-COLON software version 1.20; im3D, Turin, Italy) whose stand-alone performance has been reported elsewhere [44]. CTC data will be evaluated by a radiologist using a DR FR-CAD reading paradigm as previously reported [50]. In brief, the radiologist will examine two lists of CAD prompts, generated from the prone and supine series respectively, using both two-dimensional and three-dimensional viewing. Each CAD lesion may be rejected by the radiologist as a CAD false-positive finding, or accepted as a suspicious abnormality. To avoid missing large flat or unusual lesions, the first-reader CAD will be followed by a rapid unassisted two-dimensional interpretation, again supplemented by three-dimensional viewing for problem-solving. The radiologists reporting in the trial must have: 1) reported at least 300 colonoscopy-verified CTC studies; 2) attended a 3 day hands-on CTC screening course; and 3) have obtained a per-patient sensitivity and specificity of at least 90% during a final examination, which consists of interpreting 30 screening cases.

### CTC lesions

Diminutive polyps (5 mm or smaller) will be ignored, as their potential for malignancy is very low. For all lesions 6 mm or larger, data on malignant certainty (low, moderate, or high), size, location, and morphology will be documented. Lesion size will be measured on either multiplanar reconstructions or three-dimensional images. Morphological features (sessile, pedunculated, or flat) will be noted. Non-polypoid lesions will be defined as those with a base at least twice as long as the height. Lesion location will be described in term of the six segments of the colon: the rectum, sigmoid colon, descending colon, transverse colon, ascending colon, and cecum. Polyps 6 mm or larger detected by CTC will be verified using segmental unblinding colonoscopy. For each lesion, a true-positive CTC finding will be defined as a lesion of at least 6 mm that is found at CTC in the same or adjacent segment of the colonoscopy, with the size within 50% margin of error.

Extracolonic evaluation of the CT images will be performed in conjunction with the indicated colorectal evaluation, using standard review with soft-tissue window. Thus, there will not be active searching for extracolonic lesions. Specific notes will be made in all cases in which extracolonic lesions are found. However, only patients presenting with findings of greater potential clinical importance (that is, grade E4 according to the CT Colonography Reporting and Data System-C-RADS classification [37] and aortic aneurysms ≥4 cm) will be referred to further work-up.

### FOBT

We will use an immunological latex agglutination test (OC Sensor, Eiken, Tokyo, Japan), performed on a single stool sample with no dietary restrictions imposed on the participant, with the positivity cut-off set at 100 ng/ml. All FOBT samples collected in each study location will be shipped weekly to a single central laboratory and processed (automated processing and reading) in accordance with the manufacturer’s instructions. Participants with a positive test will be contacted by the study staff and offered an appointment date for a colonoscopy. A standard response letter will be mailed to participants with a negative result.

### Complications

Any complications that arise during screening (CTC or FS), or immediately afterward, will be reported in the examination record. Complications of CTC or FS that occur within 30 days after the procedure will be recorded through the linkage with hospital discharge records and mortality registers. All complications will be detailed according to type, timing, severity, treatment, and outcome. Information about subsequent complications will be collected through self-report telephone questionnaire administered 1 month after the examination. In addition, we will search the regional hospital discharge databases to check for any hospitalizations possibly related to screening test complications.

### Follow-up and management of polyps

#### FS

All participants will be informed about the results of their examination on the day of the procedure. If any small polyps (<10 mm) have been removed, histology assessment of tissue samples will be used to provide a definitive diagnosis and participants will be informed about the results within 2 weeks. Colonoscopy will be offered to any participants with advanced adenomas (that is, one adenoma ≥ 10 mm, or high-grade dysplasia, or villous component >20%), or with three or more adenomas of any type. Participants with a negative FS result or with hyperplastic polyps or ‘low-risk’ adenoma (that is, <2 tubular adenomas with low-grade dysplasia and smaller than 10 mm) will not undergo further follow-up.

#### CTC

If CTC reveals at least one lesion of 6 mm (C-RADS grade 2) or larger (C-RADS 3 to 4), the participant will be contacted by telephone and invited to undergo colonoscopy [37]. Participants whose examintion is inadequate according to the reporting radiologist, either because of poor bowel preparation, poor distension, or presence of artifacts, will be invited by telephone to undergo FS. Participants with no colonic lesions or with polyps smaller than 6 mm at CTC will be classified as negative, and informed of their result by regular mail. This cohort of participants will be scheduled for invitation to undergo a FOBT after 2 years. Participants with relevant extracolonic findings (that is, grade E4 and aortic aneurysms >4 cm) will be contacted by telephone and invited to the radiology unit of their nearest participating center for further investigations [37]. Extracolonic diseases that have already been diagnosed prior to CTC will be excluded from further assessments.

#### FOBT

All participants with a negative FOBT test will receive a letter notifying the result, and an indication to repeat the test after 2 years. All participants with a positive FOBT result will be invited by telephone to undergo colonoscopy.

#### Second-level colonoscopy

Second-level colonoscopy will be performed at the endoscopic unit of each participating center, using 130 cm colonoscope and air insufflation. Bowel preparation consists of a low-fiber diet starting 3 days before the examination and by the oral intake of 2 liters of polyethylene glycol solution (Moviprep; Norginey) followed by 2 liters of clear liquids the afternoon before examination. No standard protocol for sedation will be introduced. However, all centers will be required to discuss with the participant the advantages and drawbacks of performing sedated colonoscopy. Colonoscopy will be considered complete if the cecum is visualized or, in the case of failure, if a subsequent colonoscopy performed within 6 months after the index one is able to reach the cecum. The combined results of the two examinations will be included in the analysis. All detected lesions will be measured with open biopsy forceps and annotated according to their size, macroscopic appearance (sessile, pedunculated, non-polypoid, vegetating, stenosing), and location (rectum, sigmoid, descending, transverse, ascending, cecum).

#### Pathology

Histology will be defined according to the Word Health Organization criteria [54]. Advanced adenoma will be defined as an adenoma with any of the following features: size 10 mm or larger, high-grade dysplasia, or a villous component of more than 20%. Cancer will be defined as the invasion of malignant cells beyond the muscularis mucosae. Participants with intramucosal carcinoma or carcinoma *in situ* will be classified as having high-grade dysplasia. Participants will be classified on the basis of their most advanced lesion in order to determine the prevalence of pathological features. Polyp size will be classified according to the diameter of the largest polyp; for each polyp, we will use the largest measure indicated by either the endoscopist or pathologist.

### Questionnaires

#### Acceptability of the screening tests

The efficiency of CRC screening depends upon participants accepting and being able to complete the recommended procedures [55]. Therefore, we will investigate several issues related to the proposed screening tests, using questionnaires administrated at three time points to all participants in the CTC and FS groups. First, a short itemized questionnaire evaluating discomfort due to bowel preparation for the procedure will be administered immediately before CTC and FS. After completing the procedure, a second questionnaire assessing discomfort, pain, and embarrassment experienced during the procedure will be administered. A third questionnaire investigating all aspect of the screening procedure will be administered by telephone interview 1 month after the examination.

#### Reasons for participation or non-participation

Evaluation of factors associated with participation or non-participation in a new screening program is an essential component of this project. To understand the reasons for participation or non-participation, a structured questionnaire will be administered by telephone to a sample of individuals (both attendees and non-attendees) who were invited to the study 2. This will assess demographic and socioeconomic status, self-reported health status, beliefs about the benefit of screening, attitude to having regular medical control, perception of CRC risk, adoption of health-promoting behaviors (for example, physical activity, smoking habits). Factors including medical advice about CRC, knowing a close relative or friend with CRC, and experience of previous examinations for early CRC detection or for other preventive tests (for example, mammography/PAP test for women, prostate testing for men) will be evaluated. Included items are drawn from an existing questionnaire [56]. Both attendees and non-attendees will be asked to indicate their main reason for accepting or refusing screening. Non-attendees will be asked to indicate factors that might possibly induce them to undergo screening in the future, and to answer questions investigating their knowledge of aspects of the screening process as described in the leaflet (for example, bowel preparation, duration of examination).

#### Cost-effectiveness analysis

This trial includes a full economic evaluation to examine the relative cost-effectiveness of CTC screening compared with FS screening and with no screening. Cost-effectiveness analysis will be evaluated by incorporating the trial data into a previously validated screening model [33]. The advantage of this analysis is that we will collect cost data prospectively as part of the present screening trial, including the direct costs (related to provision of a diagnostic screening service) and the private costs to subjects of participation in the study, such as travel costs, out-of-pocket expenses, and costs of productivity loss. Direct costs will include cost of investigating the CTC colorectal and extracolonic findings, and the costs of screening-induced complications. In detail, the yearly CTC cost will be determined by separately collecting data relative to the following costs: 1) medical/non-medical staff; 2) initial and maintenance costs for CTC and colonoscopy equipment; 3) disposable material for CTC and colonoscopy; 4) hospital furniture for CTC and colonoscopy; 5) histological analysis of colonoscopy-detected lesions; 6) other costs (for example, investment in IT). These yearly costs will therefore be divided by the estimated number of CTC screenings to be performed in 1 year, in order to calculate the cost of each CTC screening test. The effect of CTC examination on the quality of life (QOL) of the participants will be indirectly estimated by the questionnaires assessing the degree of satisfaction and anxiety, which will be administered after CTC screening completion. It is beyond the scope of this study to assess the QOL of any participants diagnosed with cancer, because of the very small number of cancers expected in each arm. Furthermore, the cost-effectiveness analysis will take into account the effects on health-related QOL (or health status) from the process of screening for CRC, such as pain, physical discomfort, or feelings of anxiety or emotional distress. This analysis may provide important information for larger-scale CRC screening programs using CTC.

### Ethics approval

Approval for the study has been granted by the local Ethics Review Committee in each clinical site: Comitato Etico dell'Azienda Ospedaliera Universitaria S. Luigi Gonzaga di Orbassano; AO Città della Salute e della Scienza di Torino; Comitato Etico Interaziendale AOU ‘Maggiore della Carità`,’ ASL Biella, ASL Novara, ASL Vercelli, ASL VCO; Comitato Etico Regina Margherita-Sant'Anna-Ordine Mauriziano; amd Comitato Etico della Provincia di Verona.

### Data analyses

The primary endpoint of the study 1, the detection rate of the screening test, is defined as the proportion of screened subjects with advanced neoplasia divided by the total number of screened subjects. Advanced neoplasia comprises all cancers and advanced adenomas together. The most advanced detected lesion per screened subject will be used to calculate the detection rate. The overall prevalence of polyps of any histology will also be calculated. Detection rates and detection rate differences will be calculated, and all proportions will be reported with 95% confidence interval (CI). Differences in proportions between screening strategies will be calculated using the χ^2^ test. Multivariate logistic regression models with advanced neoplasia as a function of sex, center, and screening test will be used to analyze the differences in detection rate between screening tests. Odds ratio (OR) and its 95% CI will be used as a measure of the association between detection and the variables under evaluation. As a secondary analysis, we will calculate referral rate for each screening test, defined as the proportion of screening tests referred for colonoscopy because of an abnormal result. For the comparison topic, we will use the number of diagnostic colonoscopies induced by each screening test as a proxy for resource utilization and costs.

The primary endpoint of study 2, the participation rate for the screening test, is defined as the number of participants undergoing the screening test relative to the total number of invitees. The χ^2^ test will be used to test if the CTC group achieves a statistically significant difference in participation compared with the FS group. Multivariate models will be fitted to the data, with participation as a function of sex, screening test, and sociodemographic characteristics. All statistical tests will be two-sided and considered statistically significant at *P* < 0.05.

### Sample size

#### Comparison with endpoint of screening detection (study 1)

Based on the data from the Screening Colon Retto (SCORE3) trial [53], the overall prevalence of advanced neoplasia (advanced adenomas and CRC) in the target age range is assumed to be 5% at FS screening. By including 5,000 participants in the study (2,500 in each screening group), we will achieve a statistical power of 80% to detect a 2% difference in detection rate between the FS and CTC groups (5% versus 7%), using the conventional 5% (two-tailed) level of statistical significance. Assuming an adherence to the study of about 25%, we plan to invite 20,000 eligible individuals. This assumption is based on data from the SCORE3 trial [53].

#### Comparison with endpoint of adherence to screening invitation (study 2)

We plan to invite 1,000 individuals for screening with FS, under the assumption that 25% of those invited will accept the invitation [53]. Another 1,000 individuals will be invited for screening with CTC. Thus, it will be possible to detect as statistically significant an absolute difference in participation rates of more than 5% with a one-tailed test, setting the value of the alpha error equal to 5% (power = 80%).

## Discussion

FS is currently recommended for population screening programs in Italy, and is used in several regions [57]. CTC is a possible alternative to FS. To date, prospective data about the effectiveness (CRC incidence/mortality reduction) of CTC in comparison with FS for population-based screening are lacking. Moreover, several issues concerning CTC implementation as a mass screening program deserve to be addressed: patient compliance with CTC (implying reduced bowel preparation), radiologist reading times, radiologic workload, organizational impact, and economic costs. Randomized trials are clearly needed to test this novel screening approach and develop infrastructures capable of maximizing the advantages and minimizing the burden of CTC use in screening, favoring its feasibility and cost-effectiveness.

In Europe, several RCTs on the use of CTC as a CRC screening strategy have been implemented or are about to be implemented [25,40]. In these studies, CTC is compared with FOBT screening or colonoscopy as a first-level test. Our results and those of the aforementioned studies will provide national and regional health system authorities with crucial information and experience on the utility of CTC technology in CRC screening programs.

This RCT is designed to compare the detection rate of CTC for advanced neoplasia versus FS. Detection of advanced neoplasia (measured in each screening arm as the detection rate of advanced neoplasia among all participants) has been universally adopted as a crucial endpoint when validating CRC screening techniques [58]. One challenge of a trial that has detection as the primary endpoint is that the outcome assessment can be systematically influenced by selection and/or confounding biases. A RCT does not guarantee comparable groups, because selection and confounding biases may arise as a result of human interference in the recruitment procedures. For example, participants who agree to receive CTC may be systematically different from participants in the FS arm because of medical or family histories or lifestyle choices that may be associated with a different likelihood of colorectal adenomas at screening [59]. This may lead to systematic differences in the presence of advanced neoplasia between the screening arms, affecting the results of the study and their validity. Thus, we have attempted to reduce this form of bias by using a design in which only participants without strong preferences for one screening test will be randomized to the screening arms. In fact, participation in the trial is restricted to individuals who consent to randomization. Although this approach avoids confounding and should allow scientifically accurate conclusions about screening detection, it also hinders recruitment, and precludes the possibility of evaluating adhesion to CTC invitation in comparison with that to FS. However, adhesion to screening invitation is a crucial determinant of the effectiveness of a screening program. Consequentially, the evaluation of participation in a screening program should be in place before any program implementation has begun. To achieve this objective, we designed a second RCT, in which eligible individuals are randomized without consent to receive one of the two screening tests. Furthermore, this second trial makes it possible to collect data on reasons for participation or non-participation. Understanding the reasons behind participation and non-participation can be of help in the development of interventions aimed at increasing uptake of screening for CRC.

When comparing FS and CTC as CRC screening strategies, an important issue needs to be considered. FS-detected polyps 6 mm or smaller can be removed immediately, whereas such lesions are usually ignored during CTC screening. Because the long-term implications of removing diminutive polyps are unknown, long-term follow-up of very large samples would be required to estimate the protection of CTC screening against death. To our knowledge, no such trials are planned or ongoing. However, our study will provide indirect evidence of the long-term benefit from CTC screening by comparing the detection rates for CTC versus FS and assuming long-term efficacy of FS screening consistent with the results from the SCORE trial [9]. Of note, the SCORE trial evaluated the protection for FS screening over time in the same screening setting.

One more issue we kept in mind while planning the trial is that overall costs of CTC screening could be affected by uncritical reporting of extracolonic findings [37]. Whether the visualization of extracolonic structures and potential pathology is a real benefit or a disadvantage is still under debate. On the one hand, CTC may incidentally identify asymptomatic malignant diseases or other clinically important conditions, thus possibly reducing mortality. On the other hand, CTC may reveal numerous findings of no clinical relevance, which could result in costly additional diagnostic examinations with an overall negative impact on cost-effectiveness [39]. However, in some cases, detection of relevant extracolonic findings such as abdominal aortic aneurysms (AAA) and extracolonic masses at an early stage may be beneficial. According to one study, the net effect from detection of both AAAs and extracolonic cancers appears to be positive in term of life-years gained and overall cost-effectiveness [38]. Furthermore, cost-effectiveness analyses have also shown that screening for AAA provides high clinical efficacy at a relatively low cost [60]. Based on these considerations, we decide to report and follow only potentially important extracolonic findings such as abdominal and pelvic masses, and AAA larger than 4 cm, which represent only a minority of all extracolonic findings. This study will provide new knowledge on this topic through a separate analysis of the additional costs created by referring patients wtih important extracolonic findings for second-level tests.

In this trial, we are implementing a telediagnosis model for CTC reading, which consists of performing the examinations in different regional hospitals and sending data via telematic infrastructures to a central unit where the CTC examinations are interpreted by experienced radiologists. It is hoped that this organizational model will ensure high diagnostic quality, as it enables reporting to take place in a protected environment by a board of dedicated radiologists with certified experience in CTC. The use of a telediagnosis model may also favor adherence to CTC invitation thanks to the local availability of radiological centers that can perform examinations. A comprehensive evaluation on cost-efficiency of a telediagnosis model for CRC screening by CTC will include all these aspects.

In this trial, we are using the DR FR-CAD reading paradigm. In our preliminary work, we showed that this new reading modality is just as accurate as second reader CAD, but is less time-consuming [51]. It is hoped that this RCT will confirm the results of these preliminary findings.

In summary, this RCT will assess the efficacy of a CRC prevention program with CTC as a mass screening strategy in some regions of Italy, providing information about adherence, detection rate, and costs of this test compared with FS. In addition, the collection of data for cost-effectiveness analysis as part of this RCT will help to capture important aspects of CTC screening, such as adverse effects on QOL, resource use, and indirect costs. Thus, this trial will provide relevant information to decision-makers who need to consider evidence of economic value along with clinical efficacy when making decisions on resource allocation.

## Trial status

The trial started recruitment in December 2010. At the time of writing, 2,000 participants had been enrolled in the study overall.

## Abbreviations

AAA: Abdominal aortic aneurysms; CAD: Computer-aided detection; DR FR-CAD: Double-reading first-reader computer-aided detection; C-RADS: CT Colonography Reporting and Data System; CRC: Colorectal cancer; DICOM: Digital Imaging and Communications in Medicine; FOBT: Fecal occult blood test; iFOBT: Fecal immunochemical test; gFOBT: Guaiac fecal occult blood test; FS: Flexible sigmoidoscopy; CT: Colonography; CTC: RCTRandomized clinical trial; IT: Information technology; ICT: Information communications technology; QOL: Quality of life.

## Competing interests

This research is funded by the Piedmont region (Italy) and im3D SpA, Torino, Italy. Two authors (LC and AB) are employees of im3D, and one author (GI) is a research consultant for im3D. CH, CS, NS, DR, SM are not employees or consultants for im3D, and have control of any data and information that might present a conflict of interest for those authors who are employees or consultants for im3D.

## Authors’ contributions

DR, CS and NS conceived of the study, participated in its design and coordination, and helped to draft the manuscript. CS and NS are responsible for analysis and interpretation of the data. GI contributed to study design and coordination, was responsible for the radiologists' training, and contributes to data acquisition, and drafting of the manuscript. LC contributed to study conception and design, participates in to data acquisition and statistical analyses, and helped to draft the manuscript and CH is responsible for the cost-effectiveness analysis, contributes to the analysis and interpretation of data, and helped to draft the manuscript. AB participated in the study design and helped to draft the manuscript. SM participated in the study and protocol development, and helped to draft the manuscript. All authors are responsible for the study design and revision of the manuscript. All authors have read and approved the manuscript.
